# Blockade of surface bound TGF-β abrogates Treg suppression of effector T cell function within the tumor microenvironment

**DOI:** 10.1186/2051-1426-2-S3-P192

**Published:** 2014-11-06

**Authors:** Sadna Budhu, David Schaer, Yongbiao Li, Alan Houghton, Samuel Silverstein, Taha Merghoub, Jedd D Wolchok

**Affiliations:** 1 Immunology, Memorial Sloan Kettering Cancer Center, New York, NY, USA; 2 Cancer Immunobiology, ImClone Systems, New York, NY, USA; 3 Research Engineering, Memorial Sloan Kettering Cancer Center, New York, NY, USA; 4 Physiology and Cellular Biophysics, Columbia University Medical Center, New York, NY, USA

## 

Regulatory T cells (Treg) play a role in suppression of anti-melanoma immunity; however, the exact mechanism is poorly understood. Through intravital two photon microcopy, we found that tumor-specific Pmel-1 effectors engage in cell-cell interactions with tumor resident Tregs. To determine if contact between Tregs and Teff hinders killing of tumor cells *in vivo*, we utilized *ex-vivo *three-dimensional collagen-fibrin gel cultures of B16 melanoma cells. Collagen-fibrin gel cultures recapitulated the *in vivo *suppression, rendering the dissociated tumor resistant to killing by *in vitro *activated antigen specific T cells. *In vivo *depletion of Tregs in Foxp3-DTR mice prior to tumor excision reversed the suppression. *In vivo *modulation of Tregs by GITR ligation had a similar effect, reducing the number of intra-tumor Tregs leading to ex-vivo tumor killing. Using neutralizing antibodies, we found that blocking TGF-β reversed the suppression. In addition, soluble factors from collagen-fibrin gel tumors do not inhibit killing suggesting that suppression is contact or proximity dependent. The CD8 T cells recovered from these gels exhibit a decrease in Granzyme B expression and an increase in expression of T cell exhaustion marker PD-1. These findings support the conclusion that intra-tumor contact with Tregs during the effector phase of the immune response is responsible for inhibiting anti-melanoma immunity in a TGF-β dependent manner shedding light into novel ways to inhibit intratumoral Tregs.

This study was supported by Swim Across America; NIH grants R01CA56821, P01CA33049, and P01CA59350 (to J.W. and A.H.); D.S. and S.B. received support from the NIH/NCI Immunology Training GrantT32 CA09149-30.

